# The First Report of Multicentric Carpotarsal Osteolysis Syndrome Caused by *MAFB* Mutation in Asian

**DOI:** 10.1155/2018/6783957

**Published:** 2018-09-16

**Authors:** Pongsakorn Choochuen, Kitiwan Rojneuangnit, Thanitchet Khetkham, Sookkasem Khositseth

**Affiliations:** ^1^Medical Student, Faculty of Medicine, Thammasat University, Bangkok, Thailand; ^2^Department of Pediatrics, Faculty of Medicine, Thammasat University, Bangkok, Thailand; ^3^Divison of Forensic Medicine, Thammasat University Hospital, Khlong Nueng, Thailand

## Abstract

Multicentric carpotarsal osteolysis syndrome (MCTO) is a rare skeletal disorder characterized by aggressive osteolysis associated with progressive nephropathy. The early clinical presentation can mimic polyarticular juvenile idiopathic arthritis. Since 2012, *MAFB* mutations have been discovered in all MCTO patients. Therefore, the early diagnosis can be made based on genetic confirmation. We report the clinical manifestation of mineral bone disease and the molecular genetic study of a Thai female adolescent with MCTO. She presented with end-stage renal disease, bilateral wrist and ankle joint deformities, and subtle facial dysmorphic features. We identified a heterozygous missense *MAFB* mutation at nucleotide 197 from C to G (NM_005461.4; c.197C>G), predicting the change of amino acid at codon 66 from serine to cysteine (p.Ser66Cys), and the mutation was absent in the parents, indicating a de novo mutation. This report confirms the previous link between *MAFB* mutation and MCTO. Her unexplained hypercalcemia after a regular dose of calcium and active vitamin D supported an important role of MafB in the negative regulation of RANKL-mediated osteoclast differentiation. Therefore, we would encourage the physicians who take care of MCTO patients to closely monitor serum calcium level and perform a genetic study as a part of the management and investigation.

## 1. Introduction

Multicentric carpotarsal osteolysis syndrome (MCTO, OMIM # 166300), also called idiopathic multicentric osteolysis with progressive nephropathy, is a rare autosomal dominant disorder characterized by progressive osteolysis, predominantly of the carpal and tarsal bones. The patients might have subtle facial features including triangular faces, micrognathia, and exophthalmos. The early clinical presentation is joint pain, which can mimic polyarticular juvenile idiopathic arthritis (JIA). Previously, several patients had been diagnosed as JIA for a few years before the development of carpal and tarsal osteolysis detected by roentgenography [1, 2]. Renal involvements including proteinuria and renal insufficiency usually develop after bone manifestations [3–5]. Therefore, patients might receive harmful and unresponsive medications before a diagnosis of MCTO [1].

In 2012, Zankl et al. [5] discovered *MAFB* mutation responsible for MCTO in 11 patients. This finding allows early diagnosis of MCTO, which guides us for the proper management, early detection of renal insufficiency, and genetic counseling for their families. We report clinical data and disease progression of a 14-year-old girl with *MAFB* mutation confirming MCTO, throughout her life.

## 2. Case Report

**Figure 1:**
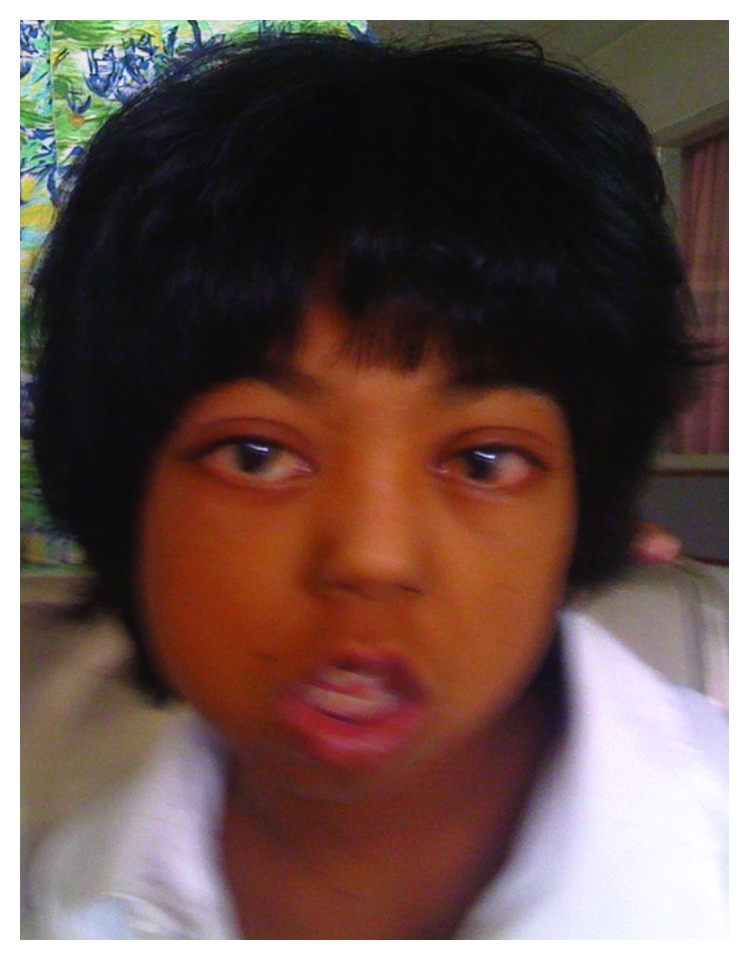
Patient's facial appearance demonstrating cloudy cornea, exophthalmos, underdeveloped ala nasi, maxillary hypoplasia, and micrognathia.

A 14-year-old girl came to Thammasat University Hospital for renal replacement therapy. She presented with end-stage renal disease (oliguria, anemia, and failure to thrive). She was the first child of healthy nonconsanguineous Thai parents. She was born at term to a 23-year-old mother and a 25-year-old father. Her prenatal and perinatal periods were uncomplicated. Her development was unremarkable until six months of age. She clawed by using her knees and elbows, instead of hands, at the age of 8 months. She was unable to walk on her feet but moved on her knees during her childhood period. She came to our hospital at the age of 12 years. Her weight was 25.4 kilograms (below the 3^rd^ percentile) and her sitting height was 70 cm (below the 3rd percentile). Her vitals were normal, except high blood pressure at 120/80 mmHg (above 99^th^ percentile for her age, sex, and height). She had pallor. Her distinctive facial features were the cloudy cornea, exophthalmos, underdeveloped ala nasi, maxillary hypoplasia, and micrognathia (Figure 1). Upper extremity deformities included shortening of arms and forearms, flexion contracture of elbows, distorted wrists, and shortening of all fingers. Deformities of lower extremities were short thighs, short-bowed legs, and flexion contracture of knee. Distorted and restricted in motion of ankles, and deformities of feet were observed. Her heart, lungs, abdomen, and neurological examination were unremarkable. Her cognitive was appropriated with age.

**Figure 2:**
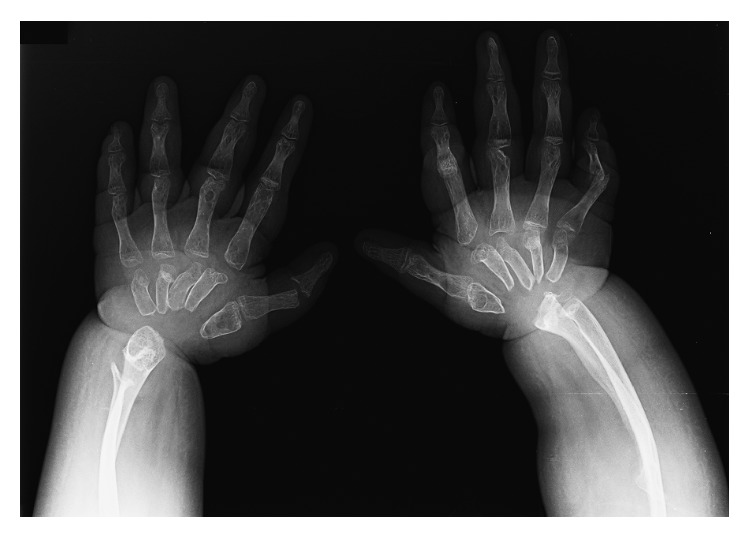
Plain hands radiograph demonstrating the absence of carpal bones and osteolysis of the proximal end of metacarpal bones as well as the resorbed distal end of ulna and radius. Severe cortical thinning of metacarpal and phalangeal bones as well as bowing of ulna and radial bones was observed.

**Figure 3:**
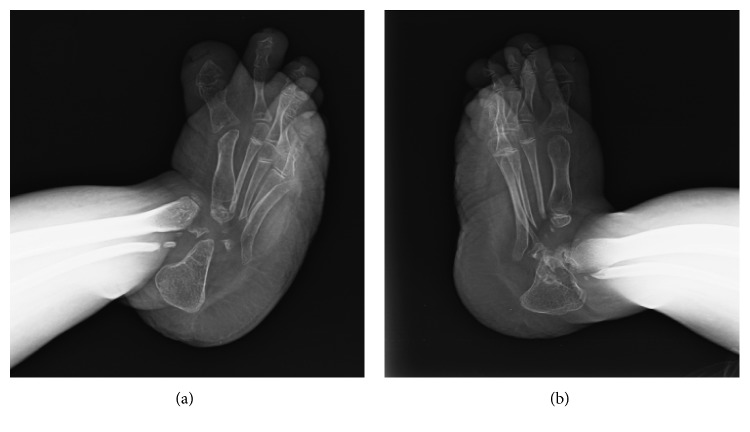
Radiographs of right (a) and left (b) ankles demonstrating the osteolysis of tarsal bones, distal tibial epiphysis, the proximal end of metatarsal bones, and the distal end of fibula. Severe cortical thinning and osteopenia of all bones as well as bowing of both tibias were observed.

Her initial investigations demonstrated blood urea nitrogen 120 mg/dL, serum creatinine 8.3 mg/dL, Na 134, K 4.5, Cl 95, HCO_3_ 11 mmol/L, phosphate 2.6, calcium 3.8 mg/dL, albumin 0.6 g/dL, alkaline phosphatase (ALP) 13.5 U/L, with parathyroid hormone (PTH) level at 94 pg/ml. Her radiography of the upper extremities showed absence of carpal bones, osteolytic lesions of metacarpal, and distal ends of ulna and radial bones (Figure 2). The radiography of the lower extremities demonstrated absence of tarsal bones, osteolytic lesions of metatarsal bones, and distal end of fibula (Figure 3). In addition, severe cortical thinning of all bones indicating osteopenia was observed (Figures 2 and 3).

Ultrasonography showed small size of both kidneys and echocardiography demonstrated left ventricular hypertrophy. The diagnosis of end-stage renal disease was made with estimated glomerular filtration rate (GFR) of 7 mL/min/1.73 m^2^. All clinical presentation and investigations were compatible with the clinical diagnosis of MCTO. She received hemodialysis followed by continuous ambulatory peritoneal dialysis and medications including erythropoietin, ferrous fumarate, 0.50 *µ*g of calcitriol, and 1400 mg of elemental calcium. Six months later, she developed generalized tonic-clonic seizure from hypercalcemia (12.4 mg/dL). Her serum chemistry demonstrated normal serum phosphorus level (3.9 mg/dL) and low serum alkaline phosphatase (4 U/L), with normal serum PTH level at 103 pg/mL. These findings indicated a marked reduction in the bone uptake of calcium after a period of calcium supplement. Calcium and calcitriol were discontinued until serum calcium returned to normal level, then only 720 mg of elemental calcium was reintroduced. Two years later, she had a slightly high serum calcium level (10.8 mg/dL), normal serum phosphorus level (3.5 mg/dL), and normal PTH level (113 pg/ml). Interestingly, during the past 2 years, we observed persistently low serum ALP levels (2.6–4.9 U/L) [6], indicating poor osteoblastic activity and limited bone formation. Thus, impaired bone mineralization from dysregulation of osteoblast and osteoclast was suspected and *MAFB* mutation might be responsible for MCTO disease in this patient.

We performed a genetic testing, *MAFB* sequencing on her and her parental blood. The genomic were isolated from peripheral lymphocytes using Puregene DNA extraction kit (Qiagen, Valencia, CA). A short region of the amino-terminal transcriptional activation domain of the *MAFB* gene, containing mutation hotspots, was amplified by polymerase chain reaction (PCR). We designed primers by using software (Primer3Plus). The forward and reverse primer sequences for PCR amplification were 5′-GCTCAAGT TCGACGTGAAGA-3′ and 5′-GTAGTTGCTCGCCATCCAGT-3′, respectively. PCR products were visualized on a 2% agarose gel and purified using DyeEx 2.0 spin kit (Qiagen, Valencia, CA). The products were then sequenced by capillary electrophoresis. This study was approved by the ethic committee of the Faculty of Medicine, Thammasat University, Thailand (MTU-EC-PE-1-005/59).

**Figure 4:**
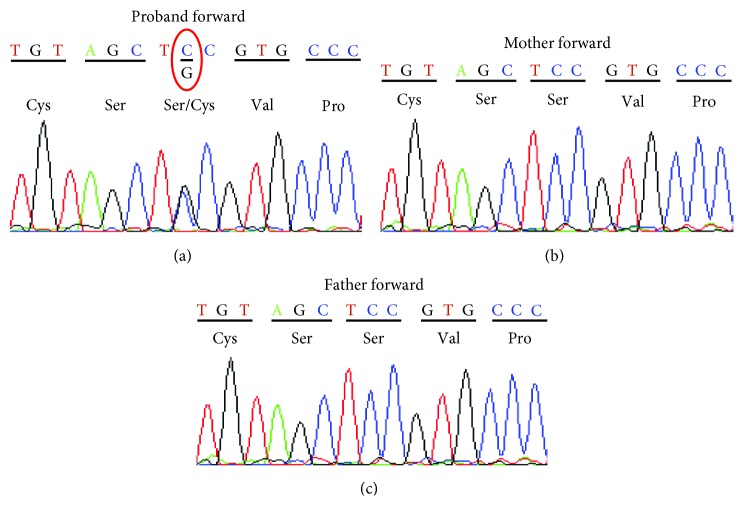
Sanger sequencing of *MAFB* gene. (a) Proband forward demonstrating a heterozygous missense mutation at nucleotide 197 from C to G (c.197C>G) that predicts the change of amino acid at codon 66 from serine to cysteine (p.Ser66Cys). The sequences of the unaffected mother (b) and the father (c) showing normal nucleotides TCC at codon 66.

We identified a de novo heterozygous missense mutation at nucleotide 197 from C to G (NM_005461.4; c.197C > G; Figure 4), predicting the change of amino acid at codon 66 from serine to cysteine (p.Ser66Cys). There was negative in both mother and father's result. This missense mutation occurred within the transactivation domain of MafB protein. This serine at the codon 66 is evolutionarily conserved among species, and in silico prediction of pathogenicity programs classify this variant as deleterious (SIFT) and probably damaging (PolyPhen).

## 3. Discussion

We report a mutation of V-maf musculoaponeurotic fibrosarcoma oncogene homolog B (*MAFB*), resulting in MCTO phenotypes. MCTO is a rare disease with unknown prevalence. This Thai girl was the first report of MCTO in Asia, confirmed by *MAFB* mutation. We also demonstrated the biochemistry of chronic kidney disease-mineral and bone disorder (CKD-MBD) in this patient, which has not been described. Although we have not performed a bone biopsy, bone demineralization or poor bone formation were highly suspected as demonstrated by unexplained hypercalcemia after a regular dose of calcium and active vitamin D in conjunction with persistently low levels of ALP, and normal PTH level. These biochemical findings might reflect progressive osteolysis observed in *MAFB* mutation.

*MAFB*, a single exon gene, is located at 20q12 and encodes the MafB protein, which functions for activation and differentiation of osteoclast and development of podocyte foot processes in renal. MafB is a negative regulator of receptor activator of nuclear factor κB ligand- (RANKL) mediated osteoclast differentiation [7]. Reduced MafB expression enhanced osteoclast activity [5], resulting in osteolysis predominantly in carpal and tarsal bones. MafB is also required for podocyte differentiation and renal tubule survival. The kidneys of mafB homozygous mutant mice display renal dysgenesis including the loss of normal foot processes of podocytes and non-cell-autonomous apoptotic cell death in tubular epithelial cells [8]. Therefore, inadequate MafB leads to abnormal renal development, renal dysgenesis, and progress to end-stage renal disease. This is consistent with the major phenotypic features of MCTO.

To date, thirty unrelated clinical MCTO Caucasian patients have been identified with 15 genotypes of *MAFB* mutations [1, 2, 5, 9], of which 25 patients had de novo mutations. All patients revealed a heterozygous missense mutation, which was in the hotspot with 18 amino acids (at codon 54–71) in transactivation domain. The amino acid at codon 66 (p.Ser66Cys) mutation identified in our Thai patient was previously reported in one Caucasian patient by Zankl et al. [5]. The identification of *MAFB* mutation at codon 66 in patients with different ethnicity provides the information of worldwide hot spot mutation.

Our patient has typical manifestations of MCTO including bone, renal, and facial features. We demonstrated unexplained hypercalcemia after a regular dose of calcium and vitamin D supplementation as well as persistently low serum ALP in an ESRD patient with *MAFB* mutation. However, the molecular mechanism of persistently low serum alkaline phosphatase in MCTO remains unknown. This information might benefit nephrologist to be aware of hypercalcemia as a consequence of using calcium phosphate binder in MCTO patient. Non-calcium-containing phosphate binder should be considered instead of calcium to prevent hypercalcemia and its complications.

Before the discovery of *MAFB* mutation by Zankl et al. [5], the diagnosis of MCTO was made based on clinical presentation and physical examination. Molecular genetic testing allows us to detect diseases earlier, which in turn prompts us to give proper management and genetic counseling. There is the evidence of incomplete penetrance in MCTO [9]. Therefore, unaffected parents should be tested for the genetic mutation.

## 4. Conclusion

We encourage clinicians to be aware of a benefit from genetic study in MCTO patient, as a part of holistic management including family planning.
